# Sexism-Related Stigma Affects Pain Perception

**DOI:** 10.1155/2021/6612456

**Published:** 2021-03-27

**Authors:** Ming Zhang, Yuqi Zhang, Zhihui Li, Li Hu, Yazhuo Kong

**Affiliations:** ^1^CAS Key Laboratory of Behavioral Science, Institute of Psychology, Chinese Academy of Sciences, Beijing 100101, China; ^2^Department of Psychology, University of Chinese Academy of Sciences, Beijing 100049, China; ^3^Department of Psychology, Dalian Medical University, Dalian 116044, China; ^4^CAS Key Laboratory of Mental Health, Institute of Psychology, Chinese Academy of Sciences, Beijing 100101, China; ^5^Wellcome Centre for Integrative Neuroimaging, FMRIB, Nuffield Department of Clinical Neurosciences, University of Oxford, Oxford OX3 9DU, UK

## Abstract

People with stigmatized characteristics tend to be devalued by others in a given society. The negative experiences related to stigma cause individuals to struggle as they would if they were in physical pain and bring various negative outcomes in the way that physical pain does. However, it is unclear whether stigma related to one's identity would affect their perception of physical pain. To address this issue, using sexism-related paradigms, we found that females had reduced pain threshold/tolerance in the Cold Pressor Test (Experiment 1) and an increased rating for nociceptive laser stimuli with fixed intensity (Experiment 2). Additionally, we observed that there was a larger laser-evoked N1, an early laser-evoked P2, and a larger magnitude of low-frequency component in laser-evoked potentials (LEPs) in the stigma condition than in the control condition (Experiment 3). Our study provides behavioral and electrophysiological evidence that sexism-related stigma affects the pain perception of females.

## 1. Introduction

Stigma is viewed as a disgraceful identity that includes a devalued social attribute [1]. People with stigmatized characteristics always are negatively viewed and devalued by others. When individuals describe the feelings of being rejected or devalued, they describe their experience with words indicating physical sensations, for example, “painful.” The negative feelings and unpleasant experiences arising from stigma not only affect an individual's social interaction [2] but also might modulate their physical perception.

Individuals with chronic pain will be vulnerable to the social consequences and negative psychology associated with stigma [3]. Stigma is internalized by people with chronic pain, who may themselves endorse stigmatizing statements [4]. Conversely, previous studies found that there might be a potential effect of stigma on pain perception. For instance, internalized stigma negatively affects psychosocial health outcomes [5]. Data from people who had low back pain suggested that stigma was associated with greater intensity of perception of chronic pain [6]. Particularly, stigmatization is the main source of social consequences for these people who have a stigmatized identity [7].

Studies focused on social pain showed a potential interaction between physical pain and social pain [8]. Eisenberger et al. [9] found that enhanced experiences of exclusion were associated with increased pain sensitivity. Social exclusion could modulate social exclusion-physical pain relation [10]. Considering that stigma can arouse negative feelings, and that perceived unpleasantness is a very important component of pain perception [11], it is reasonable to predict that stigma related to one's identity might influence individuals' pain perception. Yet, no research has directly examined the effect of stigma on stimulus-evoked pain with both behavioral and electrophysiological recordings.

In the current study, we aimed to examine whether stigma related to one's identity could affect individuals' pain perception using sexism-related paradigms, which are common ways to induce stigmatization of females [7]. Based on previous findings mentioned above, we hypothesized that sexism-related stigma could increase females' pain perception. We first examined this hypothesis in two behavioral experiments, which compared the pain sensitivity in the stigmatization condition (Experiment 1, tonic cold pain) and pain perception in the stigmatized cue condition (Experiment 2, transient laser heat pain) with those in the control condition. In order to test the stability of the effect of stigma, i.e., whether the negative effect of stigma on physical pain exists regardless of pain intensities, we used two intensities to elicit low pain and high pain in Experiment 2. Previous electroencephalography (EEG) studies found that stigmatized cues elicit early brain potentials associated with emotion processing and a late positive brain potential when individuals passively view figures, showing that stigma is a more salient cue than other negative nonstigmatized cues [12]. Instead of evaluating the brain responses elicited by stigmatized cues, we assessed the influence of the stigmatized cue on brain responses evoked by nociceptive stimuli. The laser evoked brain responses (e.g., N1, N2, and P2 components) that are highly associated with pain perception provide an objective measure of pain processing in the human brain [13]. In line with pain perception, we expected that the effect of sexism-related stigma would modulate the corresponding neural processing of noxious information reflected in some typical brain responses (e.g., the amplitudes and latencies of N1, N2, and P2 components). Therefore, we conducted an EEG experiment to investigate laser-evoked brain responses underlying the effect of stigma.

## 2. Experiment 1: The Modulation of Stigma on the Sensitivity of Tonic Pain

### 2.1. Method

#### 2.1.1. Participants

Female participants were recruited through online advertisements. People who had a history of seizures, cardiovascular disease, open cuts/sores/frostbite on their nondominant hand, or under any type of pain medication were excluded [14]. Participants were also screened, to ensure that they believed there was a foreseen negative effect coming from gender discrimination. After providing informed consent, to gain some insight into gender discrimination, participants were asked to respond to five items (adapted from a previous study [15], e.g., “If you experience gender discrimination in the workplace, it will have a negative effect on your confidence in yourself as a professional,” response from 1 = “never” to 5 = “likely”). The questionnaire terminated automatically if participants selected “never” or “rarely” for all the items. The a priori power analysis showed that a sample size of 128 allows detection of effect size (*f* = 0.30) with 80% power at an alpha of 0.05 for the design of one factor with four conditions (using G∗Power Version 3.1). A total of 129 right-handed participants in local universities (age = 21.77 ± 3.25 years, range = 18–44) passed the screening and took part in the experiment. There was one participant who is over 30 years old (i.e., 44 years old) and married. Almost identical results were obtained when this participant was excluded, suggesting the influence of age and marital status on our results would be subtle. All participants were debriefed and received ¥80 or ¥120 as compensation. All experimental procedures in this study were approved by the Institutional Review Board of the Institute of Psychology at the Chinese Academy of Sciences and were performed in accordance with the Helsinki Declaration. All participants in this study were not allowed to take part in more than one experiment.

#### 2.1.2. Procedure and Design

After completing a thorough written and verbally informed consent process, participants were asked to respond to the Pain Sensitivity Scale (e.g., “Imagine you burn your tongue on a very hot drink” [16], responses from 0 = “not at all painful” to 10 = “most severe pain imaginable”) which was demonstrated to have good reliability, with *a* = 0.92.

#### 2.1.3. Cold Pressor Test (Pretest)

Participants' baseline measures (pain threshold and tolerance) were then recorded using the Cold Pressor Test by the low-temp circulator. In the beginning, the nondominant hand was immersed for 2 minutes in warm water, maintained at 32°C to standardize the hand temperature [17]. Next, participants were instructed to insert their nondominant hands (left hand) into the cold water, for which the temperature was maintained at 2 ± 0.1°C. They were asked to report when the hand first became painful and were instructed to keep their hand in the water until they could not tolerate it anymore [18]. However, the maximum duration was set at 3 minutes [19], at which time six participants were instructed to remove their hands from the water in this session (1-2 trials each person). The latencies of pain threshold and pain tolerance were measured in milliseconds on a digital stopwatch operated by the experimenter [18]. The pain threshold was determined by measuring the amount of time (seconds) from hand insertion to the time point at which pain was reported, and pain tolerance was the time point when the participant withdrew their hands [18]. Participants orally reported their pain on two separate numerical rating scales ranging from 0 (no pain) to 10 (maximal pain) after they withdrew their hands. Five trials, with a 90-second interval between consecutive trials to prevent habituation, were taken for each participant.

#### 2.1.4. Online Job Interview

All participants were assigned to one of four conditions in alternating order (33 participants in the control group, 32 participants in the supportive group, 32 participants in the stigma group, and 32 participants in the negative group). The job interview paradigm [20] was to be used for manipulation. Participants were given the chance of an extra reward of ¥40 in addition to the standard compensation, depending on the results of the job interview paradigm in the last three groups. After providing basic demographic information, participants were asked to imagine that they were applying for a management position in an organization in the information technology sector, which employed approximately 500 people across its various departments [7]. After that, participants were asked to answer 7 interview questions (adapted from a previous study [7]), by a bogus interviewer, introduced as Xinyu Li, male, 34 years old, and with 7 years' experience in his current post [7], and the online interview was implemented by Qualtrics Panels (Qualtrics, Provo, UT, USA). After participants finished the interview questions, a quarter of the participants were asked to answer the final questions without being given any information on the extra reward and ended this task directly (the control group); a quarter of the participants got the feedback that they succeeded in the interview and received the extra reward (the supportive group); a quarter of participants got the feedback (e.g., women are generally not suitable candidates for these kinds of jobs) that they failed to get the job due to their gender and that they would not receive the extra reward (the stigma group); and the last quarter of participants got the feedback that they failed to get the job, without any reason being given (the negative group) (more details can be found in the supplementary material). Manipulation checks were included at this stage: “How likely do you think it is that Xinyu Li didn't select you due to his attitude toward women?” (responses from 1 = “extremely unlikely” to 7 = “extremely likely”); “Please indicate how you evaluated it, that is, to what extent did you experience what happened to you as pleasant or unpleasant?” (responses from 1 = “very unpleasant” to 7 = “very pleasant”).

#### 2.1.5. Cold Pressor Test (Posttest)

**Figure 1:**
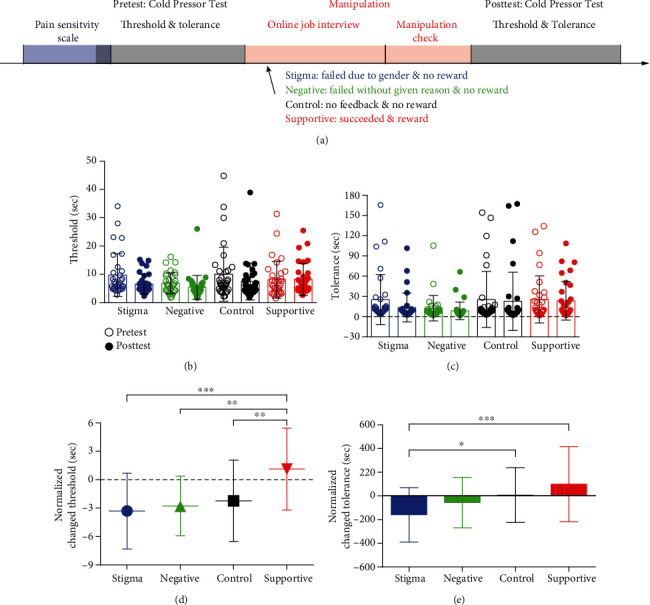
Experimental design and behavioral results in Experiment 1: (a) experimental design; (b) threshold in the pretest and posttest; (c) tolerance in the pretest and posttest; (d) the effect of condition on pain threshold; (e) the effect of condition on pain tolerance (∗∗∗*p* < 0.001, ∗∗*p* < 0.01, and ∗*p* < 0.05).

Immediately following the manipulations of stigmatization, an additional Cold Pressor Test for pain threshold and tolerance was administered, using the same procedure as at the beginning of the experiment (Figure 1(a)).

Finally, participants were given a thorough debriefing. Importantly, participants in the last three groups were told that the feedback they received in the online job interview was completely bogus and randomly assigned.

### 2.2. Results

#### 2.2.1. Group Pain Sensitivity Checks

We conducted a one-way analysis of variance (ANOVA) on the average scores of the pain sensitivity, with the condition (stigma vs. negative vs. control vs. supportive) as the between-participant factor. The results showed no significant differences in pain sensitivity across the four conditions (*F*(3, 125) = 1.28, *p* = 0.283), suggesting that participants were homogeneous in terms of pain sensitivity.

#### 2.2.2. Manipulation Checks

As intended, one-way ANOVA revealed that the manipulation affected how participants felt stigmatized (*F*(3, 125) = 18.93, *p* < 0.001, *η*_*p*_^2^ = 0.31), i.e., participants in the stigma group (5.69 ± 0.78, means M ± SD, the same hereinafter) felt stigmatized to a greater extent than participants in the control (4.69 ± 1.12) and supportive (3.94 ± 1.11) groups (*ps* < 0.001). One-way ANOVA also revealed that the manipulation affected how unhappy participants felt (*F*(3, 125) = 69.67, *p* < 0.001, *η*_*p*_^2^ = 0.63), i.e., participants in the stigma group (2.44 ± 0.76) felt much unhappier than those in the control (3.45 ± 0.87) and supportive groups (5.81 ± 1.57) (*ps* < 0.01).

#### 2.2.3. Pain Perception

The pain-related measures (threshold and tolerance, Figures 1(b) and 1(c)) were averaged separately in the pretest and posttest sessions [10]. We hypothesized that sexism-related stigma would increase pain sensitivity (i.e., reduce pain threshold and tolerance). To test this hypothesis, the changes in these measures (posttest minus pretest) were calculated to examine the effect of stigma on pain sensitivity. The changed threshold and tolerance were normalized using SPSS (by the two-step approach (We used the two-step approach for transforming nonnormally distributed continuous variables to become normally distributed, proposed by Templeton (2011). Step 1 involves transforming the variables into a percentile rank, which will result in uniformly distributed probabilities. Step 2 applies the inverse-normal transformation to the results of the first step to form variables consisting of normally distributed *z*-scores.) [21]).

*(1) Threshold*. A one-way ANOVA test on the changed threshold revealed a significant effect of condition (*F*(3, 125) = 8.04, *p* < 0.001, *η*_*p*_^2^ = 0.16). Participants in the stigma group (−3.31 ± 4.01 s) were more sensitive to pain caused by cold after they were rejected due to their gender in the online job interview, compared to those in the supportive group (1.12 ± 4.33 s, *t*(31) = −4.46, *p* < 0.001, *d* = 1.06); participants in the supportive group (1.12 ± 4.33 s) were less sensitive to pain caused by cold compared to those in the control (−2.23 ± 4.28 s) and negative groups (−2.78 ± 3.15 s, *t*(32) = 3.40, *p* = 0.005, and *d* = 0.78; *t*(31) = 3.89, *p* = 0.001, and *d* = 1.03; Figure 1(d)).

*(2) Tolerance*. One-way ANOVA on the changed tolerance revealed a significant effect of condition (*F*(3, 125) = 6.00, *p* = 0.001, *η*_*p*_^2^ = 0.13). Participants in the stigma group (−159.96 ± 229.61 s) showed worse tolerance to cold water after they were rejected due to their gender in the online job interview, compared to those in the control (6.50 ± 230.62 s) and supportive groups (98.39 ± 316.67 s, *t*(32) = −2.65, *p* = 0.054, and *d* = 0.72; *t*(31) = −4.12, *p* < 0.001, and *d* = 0.93, Figure 1(e)).

### 2.3. Discussion

In Experiment 1, individuals reported a lower pain tolerance after they had been rejected due to their gender in the job interview, which provided preliminary evidence that gender-based stigma could increase pain sensitivity in females.

## 3. Experiment 2: The Modulation of Stigma on the Perception of Transient Pain

### 3.1. Method

#### 3.1.1. Participants

The a priori power analysis demonstrated that a sample size of 34 allows detection of effect size (*f* = 0.25) with 80% power at an alpha of 0.05 for the repeated measures with two within-participant factors. A total of 33 right-handed female participants in local universities (age = 21.88 ± 2.95 years, range = 18–33) took part in this experiment, providing a 0.79 power to detect the estimated effect. All participants received ¥50 as compensation for participating in the experiment.

#### 3.1.2. Stimuli

**Figure 2:**
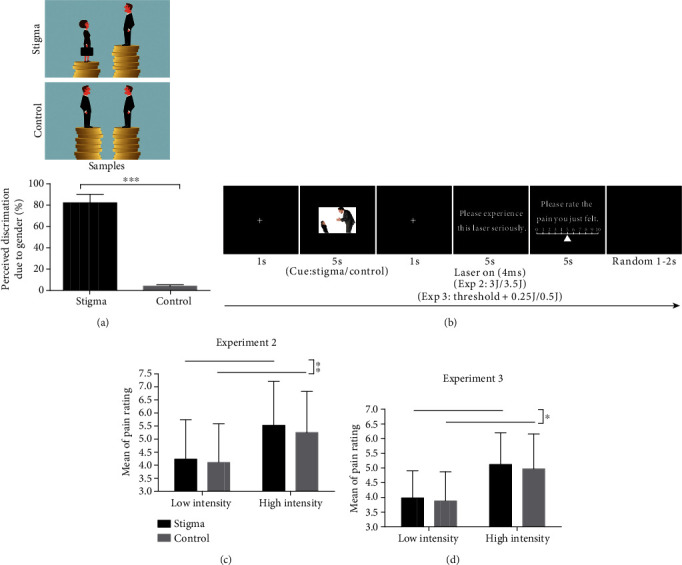
Experimental design and behavioral results in Experiments 2 and 3. (a) The material samples used in Experiment 2 and the results of stimulation assessment. (b) The experimental setup used in Experiments 2 and 3. (c) The effect of condition on ratings of pain intensity in Experiment 2. (d) The effect of condition on ratings of pain intensity in Experiment 3. (^∗∗∗^*p* < .001, ^∗∗^*p* < .01, and ^∗^*p* < .05).

A total of 57 sets of pictures were downloaded from Google and were modified by the authors' lab. There were two types of pictures in each group: (a) stigma content, with words or figures indicating devaluation of females; (b) control content, with no content relating to gender. Then, the stimulus assessment in the preexperiment had been conducted by recruiting 30 female participants (age = 21.90 ± 2.73 years, range = 19–28), who did not take part in the main experiment. They were asked to rate each picture by two questions: (1) How likely do you think it is that the content in the picture reflected an issue concerning gender discrimination or sexism? (2) Indicate how you evaluated it––that is, to what extent did the content in the picture seem to you pleasant or unpleasant (response conveyed by a slider from 0 to 100% in increments of 10%)? We used these ratings to rank the pictures and selected the top 30 pictures in the stigma condition and the last 30 in the control condition. Stigmatization ratings showed the extent to which all sets of pictures were seen as indicating sexism (stigma: 82.12 ± 8.03 vs. control: 4.11 ± 1.24; *t*(29) = 52.60, *p* < 0.001, and *d* = 13.58) (Figure 2(a)). And the pleasantness rating showed these stigmatized pictures (76.26 ± 10.04) were more unpleasant than those in the control condition (7.53 ± 6.30; *t*(29) = 31.76, *p* < 0.001, and *d* = 8.20). Finally, 30 figures rated as conveying stigma and 30 control pictures were used as the stimuli in the experiment (see Figure 2(a) for material samples).

#### 3.1.3. Nociceptive Stimulation

Nociceptive heat stimuli were generated by an infrared neodymium yttrium aluminum perovskite (Nd: YAP) laser with a wavelength of 1.34 *μ*m (Electronic Engineering, Italy). Laser pulses were delivered to a squared area (5 × 5 cm^2^) on the back of the left hand while a HeNe laser pointed to the area to be stimulated. The 4 ms pulsed laser beam was transmitted via optic fiber, and its diameter was set at approximately 7 mm (≈38 mm^2^) by focusing lenses [13]. After each stimulus, to avoid nociceptor fatigue or sensitization, the laser beam target was shifted by at least 1 cm in a random direction [13, 22]. Two different intensities (3 J and 3.5 J) were used to induce low pain and high pain, respectively. Participants were asked to report the perceived intensity of pain elicited by laser stimuli on a numerical rating scale (NRS) ranging from 0 (no pain) to 10 (pain as bad as it could be), with 4 denoting the threshold of pain [22].

#### 3.1.4. Procedure and Design

The experiment included two blocks in the same session, with 15 trials for each condition in each block (60 trials in total). The presentation of stimuli was controlled using E-Prime 2.0 (Psychological Software Tools, Inc., Pittsburgh, PA, USA). In each trial (see Figure 2(b)), a white fixation cross was first presented for 1 s, and then one of the two cues (stigma or control) was presented for 5 s randomly. After that, a white fixation cross was presented for 1 s; at the same time, a laser pulse (3 J or 3.5 J) was delivered to the dorsum of the participant's left hand. Participants were asked to perceive the pain they had just felt and were instructed to report the intensity of laser-evoked pain on a 0–10 NRS (displayed for 5 s). After this, a black background screen appeared for a random period (1–2 s), before the next trial.

### 3.2. Results

Pain ratings were analysed using the two-way repeated-measure ANOVA with condition (stigma/control) and intensity (low/high) as within-participant factors. The ANOVA revealed that both the main effects of condition and intensity were significant: *F*(1, 32) = 8.12, *p* = 0.008, and *η*_*p*_^2^ = 0.202 and *F*(1, 32) = 163.84, *p* < 0.001, and *η*_*p*_^2^ = 0.837, respectively. The pain rating in the stigma condition (4.88 ± 2.28) was significantly higher than that in the control condition (4.68 ± 2.13) at both laser intensity levels (*t*(32) = 2.85, *p* = 0.008, and *d* = 0.09; Figure 2(c)). The pain rating at the high-intensity level (5.39 ± 2.16) was significantly higher than at the low-intensity level (4.17 ± 2.08) in both conditions (*t*(32) = 12.86, *p* < 0.001, and *d* = 0.58). The interaction of condition and intensity was not significant (*F*(1, 32) = 0.91, *p* = 0.347).

### 3.3. Discussion

In Experiment 2, we found that the stigmatized cue influenced individuals' pain perception to nociceptive laser stimuli with fixed intensity; that is, individuals felt more pain from these nociceptive stimuli in the stigma condition. Individuals' different performances in stigma and control conditions reflected a modulation effect of stigma on pain perception.

## 4. Experiment 3: The Modulation of Stigma on Pain-Evoked Brain Responses

### 4.1. Method

#### 4.1.1. Participants

The a priori power analysis demonstrated that a sample size of 34 allows detection of effect size (*f* = 0.25) with 80% power at an alpha of 0.05 for the repeated measures with two within-participant factors. A total of 35 right-handed female participants in local universities (age = 21.29 ± 2.48 years, range = 18–28) took part in the experiment. All participants received ¥120 as compensation.

#### 4.1.2. Procedure and Design

The experimental materials and nociceptive stimulation were the same as those in Experiment 2. The task was the same as in Experiment 2, but including 120 trials in four blocks. Before data collection, the threshold of pain for each participant was measured by increasing the stimulus energy in steps of 0.25 J from 2.25 J. The lower energy stimulus (threshold intensity plus 0.25 J) and the higher energy stimulus (threshold intensity plus 0.5 J) were used for the experiment.

#### 4.1.3. EEG Data Collection

Participants were seated in a comfortable chair in the temperature-controlled lab, and a curtain was used to block the participants' view of their left forearm [23]. Electroencephalogram signals were recorded using ANT Neuro system with 64 standard Ag/AgCl electrodes on a WaveGuard cap based on the international 10–20 system and eego™ mylab amplifier (eemagine Medical Imaging Solutions GmbH, Berlin, Germany). A common CPz reference was used, with the sampling rate at 1,000 Hz. Impedance levels of all channels were kept below 20 k*Ω*.

#### 4.1.4. EEG Data Analysis

EEG data were analyzed using EEGLAB (2019) in MATLAB (2017b). EEG data were band-passed filtered at 1–30 Hz, using a Hamming windowed sinc finite impulse response (FIR) filter. EEG epochs were extracted using a window analysis time of 1,500 ms (500 ms prestimulus and 1,000 ms poststimulus) and baseline-corrected using the prestimulus interval. There were 29.83 ± 0.51 trials in the stigma-low condition, 29.83 ± 0.56 trials in the stigma-high condition, 29.94 ± 0.24 trials in the control-low condition, and 29.74 ± 0.61 trials in the control-high condition after the manual rejection of trials with gross artifacts. Trials contaminated by eyeblinks and movements were corrected using an independent component analysis (ICA) algorithm based on the two criteria used in a previous study [23].

For each participant, epochs corresponding to the four conditions (stigma-low, stigma-high, control-low, and control-high) were averaged as time-locked to the onset of laser stimuli. Peak latencies and amplitudes of the N1 wave (at C4-Fz, 130–200 ms), and the N2 and P2 waves (at Cz-nose, 180–230 ms for N2, 300–370 ms for P2) were obtained from the single-participant average waveforms [20, 22, 24], which were further compared using a two-way repeated-measure ANOVA with condition and intensity as within-participant factors.

Both phase-locked and non-phase-locked brain responses contained important information related to the cortical processing of nociceptive stimuli [25]. We further performed a time-frequency analysis to explore both phase-locked and non-phase-locked information evoked by laser stimuli. The time-frequency distribution (TFD) of single-trial EEG time course was obtained using a windowed Fourier transform (WFT) with a fixed 200 ms Hanning window [23, 25]. The time-frequency estimate at each point on the time-frequency plane extending from −500 to 1,000 ms (in steps of 1 ms) in the time domain and from 1 to 30 Hz (in steps of 1 Hz) in the frequency domain [23]. Using the subtraction approach, the spectrograms obtained were baseline-corrected (reference interval: −400 to −100 ms relative to laser stimulus onset to avoid the influence of spectral estimates biased by windowing poststimulus activity and padding values) at each frequency [25]. Previous studies showed that laser stimuli elicited a large phase-locked laser-evoked potential (LEP) and a clear non-phase-locked alpha ERD response (alpha-ERD), which were, respectively, maximal at central and occipital electrodes [13, 23, 26–28]. Therefore, two time-frequency regions of interest (ROIs), LEP (180–380 ms and 1–9 Hz) and alpha-ERD (600–1,000 ms and 8–13 Hz), were defined on the baseline-corrected TFDs. LEP and alpha-ERD magnitudes were, respectively, measured at electrodes displaying maximal responses based on their respective scalp topographies, i.e., at the central electrode for LEP and at occipital electrodes (PO3, PO4, PO7, PO8, O1, and O2) for alpha-ERD, which also were reported in the previous study [23]. The mean magnitudes of LEP and alpha-ERD were computed for each subject and condition and were compared using a two-way repeated-measures ANOVA with condition and intensity as within-participant factors.

### 4.2. Results

#### 4.2.1. Behavior Performance

Pain ratings were analysed by two-way repeated-measure ANOVA with condition and intensity as within-participant factors. It revealed that both the main effects of condition and intensity were significant: *F*(1, 34) = 6.89, *p* = 0.013, and *η*_*p*_^2^ = 0.168 and *F*(1, 34) = 91.73, *p* < 0.001, and *η*_*p*_^2^ = 0.730, respectively. The pain rating in the stigma condition (4.55 ± 1.89) was significantly higher than that in the control condition (4.43 ± 1.92) at both intensity levels (*t*(34) = 2.63, *p* = 0.013, and *d* = 0.06; Figure 2(d)). The pain rating at the high-intensity level (5.05 ± 1.94) was significantly higher than that at the low-intensity level (3.93 ± 1.69) in both conditions (*t*(34) = 9.56, *p* < 0.001, and *d* = 0.62). The interaction of condition and intensity was not significant (*F*(1, 34) = 0.208, *p* = 0.651).

#### 4.2.2. EEG Results

**Figure 3:**
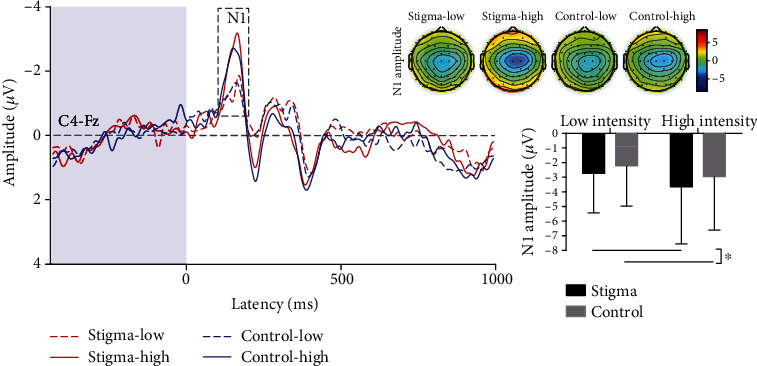
Laser-evoked potentials (N1 wave) in four experimental conditions. N1 amplitudes were significantly higher in the stigma condition than those in the control condition (^∗^*p* < 0.05). Scalp topographies of N1 waves in four conditions were displayed at their peak latencies.

Two-way repeated-measure ANOVA also revealed that there were significant main effects of condition and intensity for N1 amplitude (*F*(1, 34) = 4.46, *p* = 0.042, and *η*_*p*_^2^ = 0.116; *F*(1, 34) = 5.38, *p* = 0.027, and *η*_*p*_^2^ = 0.137). N1 amplitude in the stigma condition (−3.19 ± 3.37 *μ*V) was significantly higher than that in the control condition (−2.60 ± 3.23 *μ*V) at both intensity levels (*t*(34) = 2.40, *p* = 0.02, and *d* = 0.18; Figure 3). N1 amplitude at the low-intensity level (−2.48 ± 2.72 *μ*V) was significantly lower than that at the high-intensity level (−3.31 ± 3.77 *μ*V) in both conditions (*t*(34) = −2.84, *p* = 0.006, and *d* = 0.25; Table 1). There was no significant interaction between condition and intensity (*F*(1, 34) = 0.17, *p* = 0.685). There was no significant condition effect for N1 latency (*p* > 0.05; Table 1).

**Figure 4:**
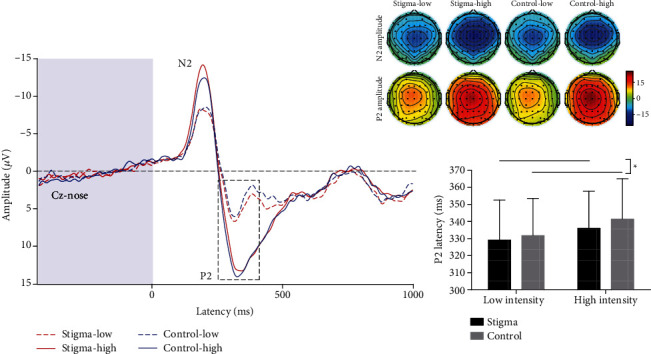
Laser-evoked potentials (N2 and P2 waves) in four experimental conditions. P2 latencies were significantly earlier in the stigma condition than in the control condition (^∗^*p* ≤ 0.05). Scalp topographies of N2 and P2 waves in four conditions were displayed at their peak latencies.

Two-way repeated-measure ANOVA revealed significant main effects of condition and intensity for P2 latency (*F*(1, 34) = 4.48, *p* = 0.042, and *η*_*p*_^2^ = 0.116; *F*(1, 34) = 7.83, *p* = 0.008, and *η*_*p*_^2^ = 0.187). The P2 latency in the stigma condition (333 ± 23 ms) was significantly earlier than that in the control condition (337 ± 23 ms) at both intensity levels (*t*(34) = −1.97, *p* = 0.05, and *d* = 0.17; Figure 4). The P2 latency at the low-intensity level (330 ± 23 ms) was significantly earlier than that at the high-intensity level (339 ± 23 ms) in both conditions (*t*(34) = −3.21, *p* = 0.002, *d* = 0.37). There was no significant interaction between condition and intensity (*F*(1, 34) = 0.40, *p* = 0.529). There was no significant condition effect for N2 latency, N2 amplitude, and P2 amplitude (*ps* > 0.05; Table 1).

**Figure 5:**
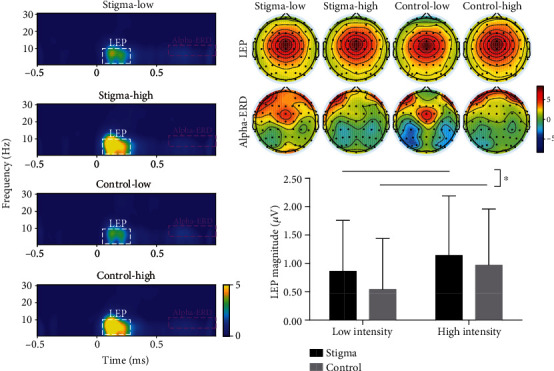
Laser-evoked modulations of EEG responses in four experimental conditions. LEP magnitude in the stigma condition was significantly larger than that in the control condition (^∗^*p* < 0.05). No significant difference was observed in alpha-ERD magnitude measured at occipital electrodes (PO3, PO4, PO7, PO8, O1, and O2) between the sigma and control conditions. Scalp topographies of response magnitudes in four conditions were displayed for each time-frequency feature (i.e., LEP and alpha-ERD).

Two-way repeated-measure ANOVA performed on time-frequency features revealed significant main effects of condition and intensity for LEP magnitude (180 ms–380 ms and 1–9 Hz, Cz-nose; *F*(1, 34) = 5.23, *p* = 0.029, and *η*_*p*_^2^ = 0.133; *F*(1, 34) = 8.98, *p* = 0.005, and *η*_*p*_^2^ = 0.209), but no significant interaction effect (*F*(1, 34) = 0.716, *p* = 0.403). LEP magnitudes in the stigma condition (1.00 ± 0.98 *μ*V) were significantly larger than those in the control condition (0.76 ± 0.96 *μ*V) at both intensity levels (*t*(34) = 2.51, *p* = 0.015, and *d* = 0.25; Figure 5). LEP magnitudes at the high-intensity level (1.06 ± 1.02 *μ*V) were significantly larger than those at the low-intensity level (0.70 ± 0.91 *μ*V) in both conditions (*t*(34) = 3.40, *p* = 0.001, and *d* = 0.37). Two-way repeated-measure ANOVA showed no significant main effects of condition and intensity, as well as their interaction for alpha-ERD magnitude (600 ms–1,000 ms and 8–13 Hz, at occipital electrodes, i.e., PO3, PO4, PO7, PO8, O1, and O2; *F*(1, 34) = 0.33, *p* = 0.57; *F*(1, 34) = 0.98, *p* = 0.33; *F*(1, 34) = 0.31, *p* = 0.58; Table 1).

### 4.3. Discussion

There was a significant effect of stigma on neurobiological responses to noxious stimuli: N1 amplitude and P2 latency in the time domain and LEP magnitude in the time-frequency domain were significantly influenced by the stigmatized cues. We provided neurophysiological evidence showing the effect of stigma on neural processing associated with pain perception.

## 5. General Discussion

The present study tested the hypothesis that sexism-related stigma could increase pain perception. We found that individuals showed reduced pain threshold/tolerance in the Cold Pressor Test (Experiment 1) and increased pain ratings to nociceptive laser stimuli with fixed intensity (Experiment 2). Moreover, with EEG recordings, we found a significant effect of stigma on neurobiological responses to noxious stimuli: N1 amplitude and P2 latency in the time domain and LEP magnitude in the time-frequency domain were significantly influenced by the stigmatized cues (Experiment 3). These salient observations across three experiments provided strong evidence that sexism-related stigma increased female individuals' pain perception.

Pain experience can be divided into the sensory, the affective, and the cognitive components [11]. Considering that stigmatization is a harmful experience since it is based on an attribute considered disgraceful [7], we inferred that the effect of sexism-related stigma on pain perception might be due to the modulations of affective (e.g., emotion) and cognitive (e.g., attention) components of pain.

### 5.1. Emotion Related to Stigma Modulated the Perception of Pain

Emotions modulate the activities in circuits related to pain encoding and shape the neural mechanisms involved in the encoding of pain [29]. Generally, individuals felt more pain in the negative emotion status, which indicated that negative mood increases pain perception. Stigmatized cues were harmful stimuli, which entail devalued attribution and pervasive negative treatment for these stigmatized individuals. These sexism-related cues tend to arouse females' negative emotions and negative behavioral responses [7].

In the present study, stigmatization and stigmatized cues included potential devalued information in relation to gender. Like previous studies, both conditioned stimuli and social information had significant effects on pain perception [30]. The information relevant to disgraceful attributes further hurt the females emotionally [2] and intensified their perception of pain. It led us to observe the phenomenon that individuals felt more pain under stigmatized cues. Moreover, females exhibited greater sensitivity than males to pain induced by the noxious stimuli (e.g., mechanical stimuli [31]). Meanwhile, compared to male patients, females showed a prevalence in the development of chronic pain (e.g., migraine [32]). These findings may be associated with the fact that females experience stigmatization based on their gender in daily life.

The stigma-related information elicited a stronger affective response [12] and seemed to further affect an individual's nociceptive-related brain responses. In fact, a previous EEG study showed stronger responses to stigmatized cues compared to nonstigmatized cues when people simply passively viewed images [12]. Correspondingly, we found an earlier laser-evoked P2 in the stigma condition than in the control condition. P2, an important component involved in the advanced stage of perceptual processing, has been demonstrated to be highly associated with emotional modulation [33]. The information related to gender discrimination was a source of negative emotion in the stigma condition compared to the control condition and further affected the processing of the subsequent noxious stimuli. The earlier perceptual component implied that the perception of pain is modulated by negative emotion related to stigma based on one's devalued identity.

### 5.2. Attention Related to Stigma Modulated the Perception of Pain

In our study, stigmatization and stigmatized cues include potential threat information that reminded females that they faced unfair treatment because of their gender. Based on their own or others' experiences of stigmatization, people with stigmatized identities can become vigilant as to whether or not they might be devalued [34]. The threat information related to themselves always alerts individuals to that threat and causes them to pay more attention automatically. Due to the modulation of attention, more processing resources will be allocated to threat information, which further intensifies individuals' perception of pain [35]. Neural activities evoked by noxious stimuli especially reflected modulation of attention in relation to widespread sexism. N1, as a type of activity prompted by nociceptive input, has been demonstrated to be easily influenced by attention [36]. The observed large laser-evoked N1 in the stigma condition indicated that stigma-related information attracted females' priority attention, which influenced the subsequent processing of noxious stimuli.

Importantly, neural activities will increase when individuals have a large mental workload involving emotion or attention [37]. In line with these findings, we observed increased LEP magnitudes with increased pain perception after the stigmatized cues, reflecting the combination of neural activities related to N1 and P2 components. Meanwhile, the similar scalp topographies of LEP in the time-frequency domain and the N2-P2 complex in the time domain indicated that the neural activities reflected by the N2-P2 complex corresponded to those reflected by the LEP response in the time-frequency domain [23]. The increased LEP magnitudes mean that there were stronger neurobiological responses related to pain perception in the stigma condition, which suggested modulation due to the information on sexism-related stigma. These findings in the time-frequency domain further verified the modulation of stigmatized cues in the perception of pain.

In the present study, sexism-related stigma implied devalued attributes and pervasive negative treatment, which directly hurt females' emotions and reminded them to remain vigilant against sexism. Actually, the two negative aspects of stigma were associated with females' emotion and attention separately. The observed neural responses associated with emotion and attention further suggested that stigma based on gender affects not only the affective component of pain but also the cognitive evaluation relevant to pain. Considering that stigmatized cues can arouse negative feelings and threat warnings, cognitive brain regions related to emotion and attention (e.g., the dACC [38] and PFC [39]) might contribute to the modulation of pain perception. That means activities elicited by the stigmatized cues might further interferer (increase or mediate) the brain responses to the noxious stimuli. It is interesting to focus on the neural mechanism underlying the effect of sexism-related stigma on pain perception for future study.

## 6. Limitations

Limitations in Experiments 2 and 3 might be that there is no nonstigma negative condition. Consequences of stigmatization might work via negative affect, but distinguishing the special stigma effect from negative events is more helpful in understanding the exact effect of stigma related to one's identity. Though stigma and nonstigma negative events can arouse an individual's negative emotion, the highly negative social stigma is distinguished from other emotional stimuli [7]. These two forms of negative components may have different effects on the perception of pain.

## 7. Conclusion

In conclusion, our study demonstrated that sexism-related stigma increases female individuals' pain perception, representing as the reduction of threshold/tolerance, the increase of pain rating, and the modulation of pain-evoked brain responses. It should be noted that the observed effects due to sexism-related stigma were relatively weak in the present study. Therefore, our results should be interpreted with caution. Future studies should be performed to verify our results and better understand the physiological or clinical meaningfulness of the effects raised by sexism-related stigma.

## Figures and Tables

**Table 1:** Pain perception and LEP responses in different experimental conditions.

Variables	Stigma-low	Control-low	Stigma-high	Control-high
	*M* ± SD	*M* ± SD	*M* ± SD	*M* ± SD
Pain perception	3.98 ± 0.93	3.88 ± 0.99	5.12 ± 1.08	4.97 ± 1.19
N1 latency (ms)	159 ± 21	157 ± 21	151 ± 16	150 ± 16
N1 amplitude (*μ*V)	−2.73 ± 2.71	−2.23 ± 2.75	−3.65 ± 3.91	−2.97 ± 3.65
N2 latency (ms)	208 ± 17	207 ± 17	203 ± 15	207 ± 17
N2 amplitude (*μ*V)	−10.83 ± 7.33	−11.03 ± 6.15	−17.78 ± 9.77	−16.33 ± 9.72
P2 latency (ms)	329 ± 23	332 ± 22	336 ± 22	341 ± 24
P2 amplitude (*μ*V)	8.94 ± 7.64	7.85 ± 6.93	16.21 ± 10.59	16.83 ± 11.08
LEP magnitude (*μ*V)	0.86 ± 0.90	0.54 ± 0.90	1.14 ± 1.05	0.97 ± 0.99
Alpha-ERD magnitude (*μ*V)	−0.14 ± 0.44	−0.08 ± 0.58	−0.18 ± 0.60	−0.17 ± 0.65

## Data Availability

Data are available on request.

## Supplementary Materials

Supplementary Materials There are more details for Experiment 1 in the supplementary materials, which include screening questions, items on the Pain Sensitivity Scale, and examples of online job interview tasks.
