# Supplementary material for: Sexism-Related Stigma Affects Pain Perception
Source: Neural Plast. 2021 Mar 27;2021:6612456. doi: 10.1155/2021/6612456 (PMC8019650; doi:10.1155/2021/6612456)
Supplement: Supplementary Materials — There are more details for Experiment 1 in the supplementary materials, which include screening questions, items on the Pain Sensitivity Scale, and examples of online job interview tasks. [file 6612456.f1.docx]

# Supplementary material

## Screening questions in Experiment 1

Response from 1 = “*never*” to 5 = “*likely*”

1. “If you experience gender discrimination in the workplace, it will have a negative effect on your confidence in yourself as a professional”;

2. “If you experience gender discrimination in the workplace, it will have a negative effect on your career advancement”;

3. “If you experience gender discrimination in the workplace, it will have a negative effect on your job satisfaction”;

4. “If you experience gender discrimination in the workplace, it will have a negative effect on your organizational commitment”;

5. “If you experience gender discrimination in the workplace, it will have a negative effect on your career commitment”.

## Pain Sensitivity Scale in Experiment 1

Please mark the scale with a cross on the number that is most true for you. Keep in mind that there are no ‘‘right” or ‘‘wrong” answers; only your personal assessment of the situation counts. Please try as much as possible not to allow your fear or aversion of the imagined situations affect your assessment of painfulness.

How painful would that be for you?

0 = *not at all painful*, 10 = *most severe pain imaginable*

1. Imagine you bump your shin badly on a hard edge, for example, on the edge of a glass coffee table.

2. Imagine you burn your tongue on a very hot drink.

3. Imagine your muscles are slightly sore as the result of physical activity.

4. Imagine you trap your finger in a drawer.

5. Imagine you take a shower with lukewarm water.

6. Imagine you have mild sunburn on your shoulders.

7. Imagine you grazed your knee falling off your bicycle.

8. Imagine you accidentally bite your tongue or cheek badly while eating.

9. Imagine walking across a cool tiled floor with bare feet.

10. Imagine your finger has a small cut, and salt is spilt on the wound accidentally.

11. Imagine a Chinese rose thorn pricks your fingertip.

12. Imagine you hold an ice pad for a few minutes with your bare hands.

13. Imagine you shake hands with someone who has a normal grip.

14. Imagine you shake hands with someone who has a very strong grip.

15. Imagine you pick up a hot pot by inadvertently grabbing its equally hot handles.

16. Imagine you are wearing sandals and someone with heavy boots steps on your foot. 17. Imagine you bump your elbow on the edge of a table (‘‘funny bone”).

## Cover story used in Experiment 1

This study is a part of a programme of research at the department of social and organizational psychology that involves training people to conduct job-interviews through the internet. There are 2 parts in today’s study. In the first part, please imagine that you are applying for a management function at an organization after you graduate from university. Your task is to take complete a brief CV and take part in an online interview.

[Depending on your performance in the interview, you will have the chance to enter an extra reward of ¥40 in addition to the standard reward (¥80) for participation in this study (appeared in all the groups except the control group)]

## Introduction of interviewer in Experiment 1

Your interviewer is Xinyu Li. Xinyu Li is 34 years old and has been working in his current post for 7 years. He has ample experience with face to face job interviews and is now interested in exploring the possibility of doing interviews online. Next, Xinyu Li will ask you some questions. He will type in the questions and you will be able to type in your answers in the space provided. Please try to answer each question with 2-3 sentences—to provide sufficient information, but not take too long. Please try to imagine this is a real interview for a job you want to have and answer the questions as honestly as possible.

## Job-interview questions used in Experiment 1

1. Why do you want this job?

2. What aspects of your background (education and or experience) are relevant for this post?

3. What would you say is your greatest strength that is relevant for this post?

4. What are your career goals?

5. I think that men are better at supervising others, so it is important to us that women are good team players – are you a good team player?

6. Do people find you overly emotional?

7. Do you plan to stop working when you have children?

## The decisions of the interviewer in Experiment 1

**The stigma condition:**

I have made a decision. I must say that I do not find you are suitable for this job. I find that you did not provide the most appropriate responses on crucial questions. In fact, women are generally not suitable candidates for these kinds of jobs. Since you are a woman, it would be very unlikely that you would come across as a suitable candidate.

**The negative condition:**

I have made a decision. I must say that I do not find you are suitable for this job. I find that you did not provide the most appropriate responses on crucial questions.

**The supportive condition:**

I have made a decision. I must say that I find you are suitable for this job. You had provided the appropriate responses on crucial questions. Based on your performance, you can get the similar job in the future. Congratulations!
